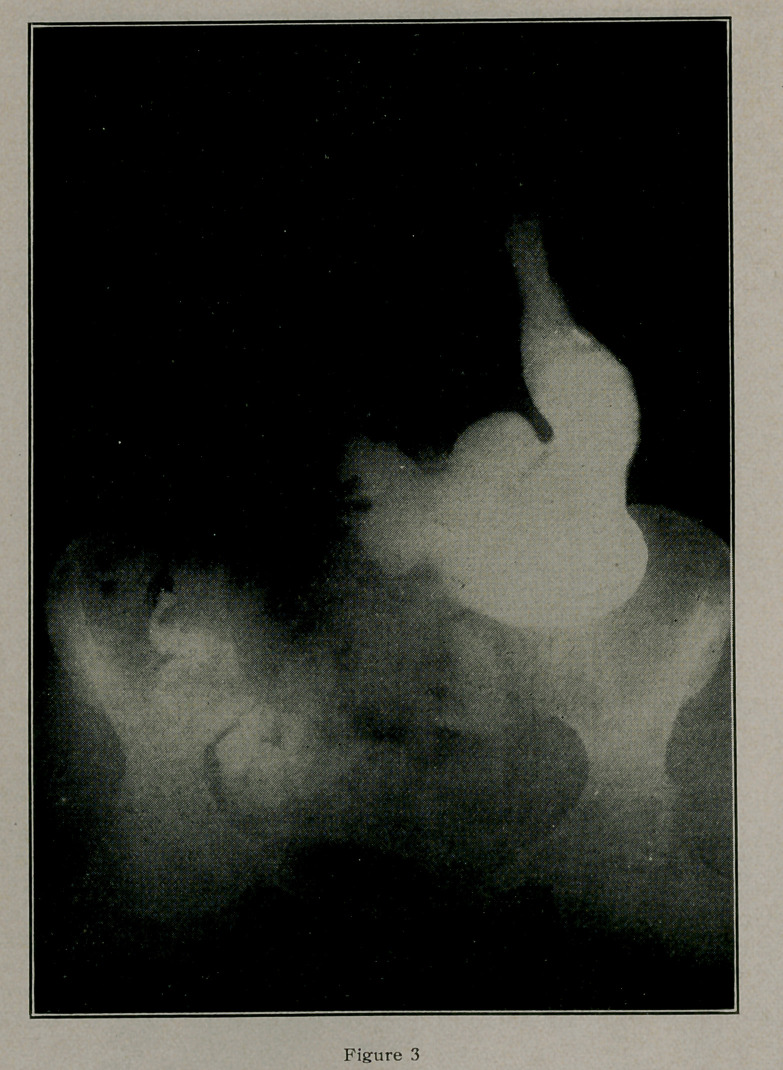# A Contribution to the Pathology of Duodenal Ulcer

**Published:** 1914-02

**Authors:** Joseph Burke

**Affiliations:** Consulting Surgeon, Emergency Hospital; Attending Surgeon, Sisters’ Hospital


### A Contribution to the Pathology of Duodenal Ulcer

BY JOSEPH BURKE, M.D., Sc.D.
Consulting Surgeon, Emergency Hospital
Attending Surgeon, Sisters' Hospital

THE clinical interest in the pathology of chronic duodenal ulcer grows in direct relation to the number of operations upon the upper abdomen. Every case seems to present an individuality all its own. These ulcers are variously situated and by the extent and character of their cicatrization often produce crippling distortions of the bowel. Thus we have annular constrictions in which the whole circumference of the duodenum, in limited extent, is affected; circular ulcers; hour-glass duodenum in which there is double constriction (cases reported by Moynihan, Mayo, Mackenzie and Burke); "kissing" ulcers, so called because they lie on opposite sides of the bowel and when the organ is empty come into contact with each other; diverticula or "pouching ulcers" described by Perry and Shaw. There is also a form of ulcer with consequent stenosis of the duodenum which I encountered in a recent operation, a form of constriction that I have never seen reported and whose rarity serves as an excuse for its detailed description. I shall designate it "tubular" constricting ulcer of the duodenum, best described by referring the reader to Fig. 1.

Mrs. M., age 46; mother of four children; family history negative. First illness at fourteen years when she suffered from hemorrhage which was diagnosed as from the lungs by one doctor, and from the stomach by another. From this time until three years ago, while always "dyspeptic," never suffered as badly as during the present illness, which began three years ago, when she suffered greatly from attacks of gas and vomiting of a sour liquid, which occurred about 4 P. M. daily and lasted sometimes until midnight. On November 5th, 1913, while shopping, was taken suddenly with pain in right hypochondrium and nausea. Pain like "knife cutting." Two hypodermics were necessary for relief of pain. "Gall stones" was the diagnosis by the attending physician.

Examination made November 22nd, 1913. Female, pale and very thin. Heart and lungs normal. Abdomen concave between ensiform and navel, but convex just below umbilicus. There is visible peristalsis over convexity, waves from left to right. Pain over gall-bladder upon deep palpation, which disappeared during artificial dilation of the stomach. Liver normal in outline. Kidneys not movable. No tenderness over appendix, no rigidity. Artificial inflation of stomach revealed dilated, gastroptotic stomach, greater curvature reaching almost to pubes;

Figure 1

visible gastric peristalsis. Reflexes normal. No oedema of feet. Urine normal; pelvis normal· Blood, secondary anaemia. Diagnosis: pylorospasm; motor insufficiency; pyloric obstruction due to duodenal ulcer; dilation of stomach.

Figure 2

ROENTGEN EXAMINATION
BY
DR. LEONARD REU.

Case No. 2068.<br>

Examination—Dec. 2, 1913.<br>

Gastro-Intestinal Tract.

Referred by<br>

Dr. Joseph Burke,<br>

1092 Main St.,<br>

Buffalo, N. Y.

X-Ray examination shows gastric dilatation, strong peristaltic and anti-peristaltic action. In standing position, the pylorus is on a level with the upper border of the sacrum. Abnormal duodenum which is of cylindrical shape and which involves the first portion of the duodenum and part of the second. This deformity of the duodenum is not like the usual

Figure 3

deformity seen in doudenal ulcer. Its outline is smooth and symmetrical.

At the end of 4 hours, the stomach contains a large amount of the bismuth meal but still shows gastric motility.

Diagnosis: Stenosis of the Duodenum.

Figs. II and III·

LEONARD REU.

Operation December 3rd, 1913; Sisters' Hospital. Upon opening the abdomen I found the stomach greatly dilated, its walls hypertrophied. The position and size corresponded to the clinical findings. About one-half inch beyond the pyloric vein, the duodenum, for about two and one-half inches appeared as a small tube constricted to about one-fourth its normal diameter, its walls indurated and somewhat rigid, the condition due to ulcer affecting the whole circumference of the bowel for about two and one-half inches. There was a cicatrix just in front of the pyloric veins on the anterior stomach wall, Fig. 1. I performed the usual posterior gastro-enterostomy by suture using a one-inch loop, the stoma being two and one-half inches, its direction from left to right on the stomach. Patient left hospital on the twelfth day after uninterrupted recovery.

1092 Main St.

---

Some General Remarks About Abstracts. Credit is given to the original source, with full name and address of author and date of journal, if accessible. We do not try to give specific credit to intermediate sources, even when we use the scissors, and do not expect credit to this Journal, except for original and editorial articles.

Some of the Abstracts are prepared by friends of the editor. We have a very convenient file of exchanges, between a radiator and a medicine cabinet. Unfortunately, when the file reaches two or three feet higher than the radiator, it gets in the way of other things. By hard and rapid work, during an afternoon we have just managed to get this file down to three feet. Spanish journals are well cared for by Major Quinton. We would like to find a translator for Italian journals, for surplus French and German journals, and for one each in some Scandinavian language, Japanese and Greek. Anyone familiar with classic Greek can read modern Greek, though he could not understand the spoken language nor even read the spoken language if it were reduced to print. This journal is of high grade professionally and the translation would be good practice for some one, with leisure. There are also a good many journals more or less limited to subjects which are outside the editor's line of practice and therefore beyond his critical ability. The editor can very readily fill sufficient space for Abstracts, in the course of a month but it is plain that a great deal of good literature is going to waste and that volunteer work would not only benefit the reviewer but the readers of the *Journal* by introducing different points of view and a wider range of interset.

## Figures and Tables

**Figure 1 f1:**
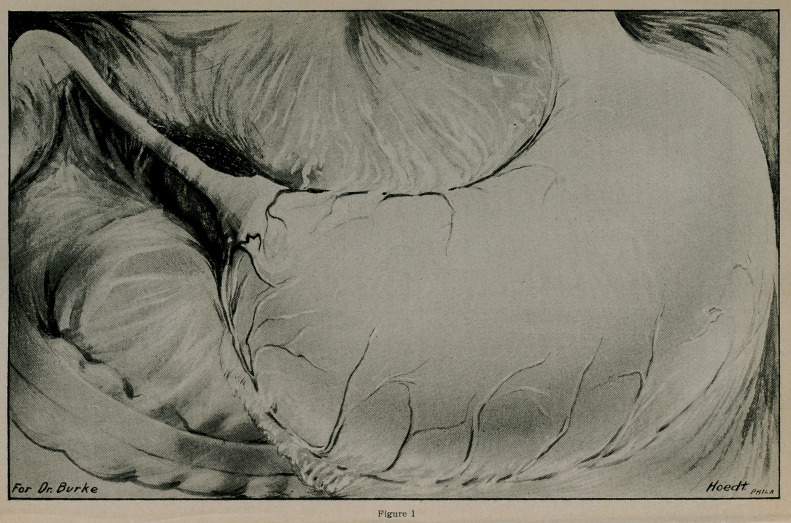


**Figure 2 f2:**
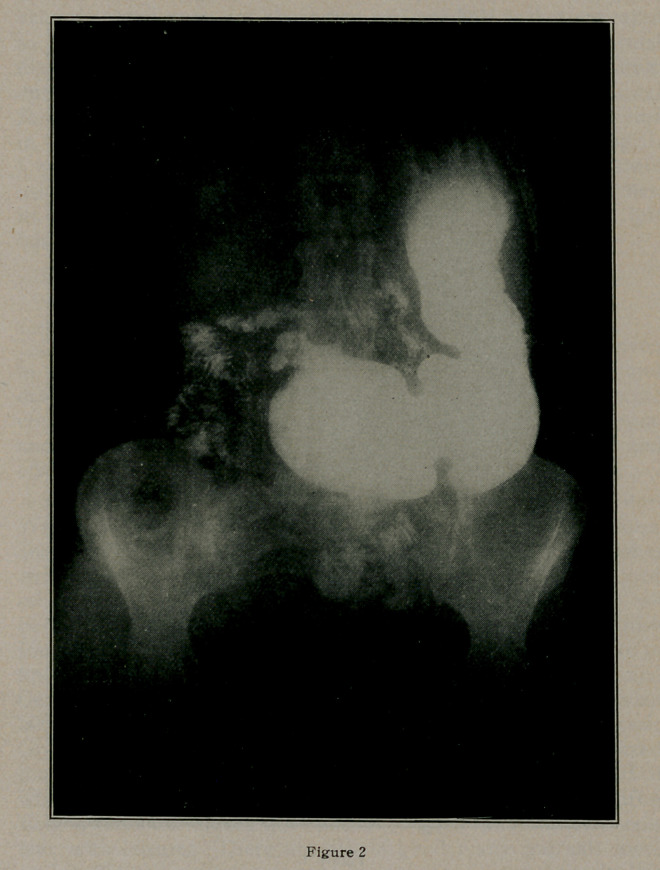


**Figure 3 f3:**